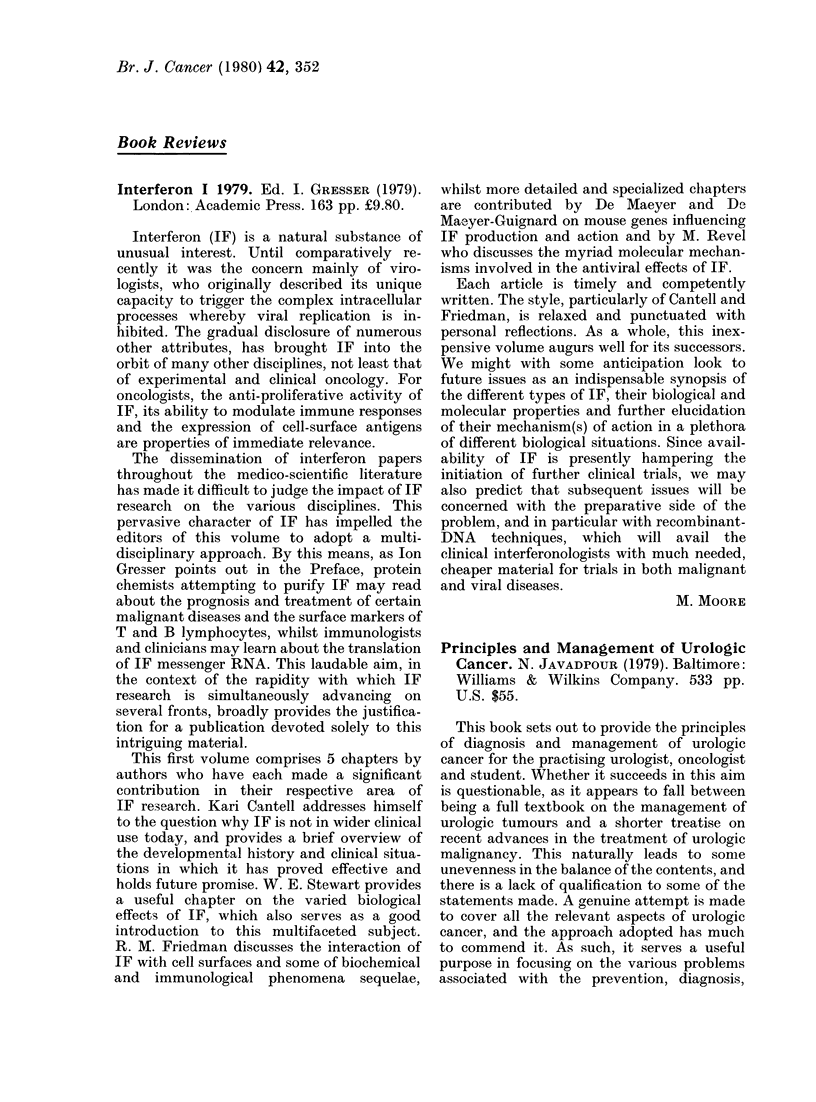# Interferon I 1979

**Published:** 1980-08

**Authors:** M. Moore


*Br. J. Cancer* (1980) **42**, 352

### *Book Reviews*

**Interferon I 1979.** Ed. I. Gresser (1979). London: Academic Press. 163 pp. £9.80.

Interferon (IF) is a natural substance of unusual interest. Until comparatively recently it was the concern mainly of virologists, who originally described its unique capacity to trigger the complex intracellular processes whereby viral replication is inhibited. The gradual disclosure of numerous other attributes, has brought IF into the orbit of many other disciplines, not least that of experimental and clinical oncology. For oncologists, the anti-proliferative activity of IF, its ability to modulate immune responses and the expression of cell-surface antigens are properties of immediate relevance.

The dissemination of interferon papers throughout the medico-scientific literature has made it difficult to judge the impact of IF research on the various disciplines. This pervasive character of IF has impelled the editors of this volume to adopt a multidisciplinary approach. By this means, as Ion Gresser points out in the Preface, protein chemists attempting to purify IF may read about the prognosis and treatment of certain malignant diseases and the surface markers of T and B lymphocytes, whilst immunologists and clinicians may learn about the translation of IF messenger RNA. This laudable aim, in the context of the rapidity with which IF research is simultaneously advancing on several fronts, broadly provides the justification for a publication devoted solely to this intriguing material.

This first volume comprises 5 chapters by authors who have each made a significant contribution in their respective area of IF research. Kari Cantell addresses himself to the question why IF is not in wider clinical use today, and provides a brief overview of the developmental history and clinical situations in which it has proved effective and holds future promise. W. E. Stewart provides a useful chapter on the varied biological effects of IF, which also serves as a good introduction to this multifaceted subject. R. M. Friedman discusses the interaction of IF with cell surfaces and some of biochemical and immunological phenomena sequelae, whilst more detailed and specialized chapters are contributed by De Maeyer and De Maeyer-Guignard on mouse genes influencing IF production and action and by M. Revel who discusses the myriad molecular mechanisms involved in the antiviral effects of IF.

Each article is timely and competently written. The style, particularly of Cantell and Friedman, is relaxed and punctuated with personal reflections. As a whole, this inexpensive volume augurs well for its successors. We might with some anticipation look to future issues as an indispensable synopsis of the different types of IF, their biological and molecular properties and further elucidation of their mechanism(s) of action in a plethora of different biological situations. Since availability of IF is presently hampering the initiation of further clinical trials, we may also predict that subsequent issues will be concerned with the preparative side of the problem, and in particular with recombinant-DNA techniques, which will avail the clinical interferonologists with much needed, cheaper material for trials in both malignant and viral diseases.

M. Moore